# Decoupling of Sleep-Dependent Cortical and Hippocampal Interactions in a Neurodevelopmental Model of Schizophrenia

**DOI:** 10.1016/j.neuron.2012.09.016

**Published:** 2012-11-08

**Authors:** Keith G. Phillips, Ullrich Bartsch, Andrew P. McCarthy, Dale M. Edgar, Mark D. Tricklebank, Keith A. Wafford, Matt W. Jones

**Affiliations:** 1 Lilly Centre for Cognitive Neuroscience, Eli Lilly and Company, Erl Wood Manor, Windlesham, Surrey GU20 6PH, UK; 2 School of Physiology and Pharmacology, MRC Centre for Synaptic Plasticity, University of Bristol, Medical Sciences Building, University Walk, Bristol BS8 1TD, UK

## Abstract

Rhythmic neural network activity patterns are defining features of sleep, but interdependencies between limbic and cortical oscillations at different frequencies and their functional roles have not been fully resolved. This is particularly important given evidence linking abnormal sleep architecture and memory consolidation in psychiatric diseases. Using EEG, local field potential (LFP), and unit recordings in rats, we show that anteroposterior propagation of neocortical slow-waves coordinates timing of hippocampal ripples and prefrontal cortical spindles during NREM sleep. This coordination is selectively disrupted in a rat neurodevelopmental model of schizophrenia: fragmented NREM sleep and impaired slow-wave propagation in the model culminate in deficient ripple-spindle coordination and disrupted spike timing, potentially as a consequence of interneuronal abnormalities reflected by reduced parvalbumin expression. These data further define the interrelationships among slow-wave, spindle, and ripple events, indicating that sleep disturbances may be associated with state-dependent decoupling of hippocampal and cortical circuits in psychiatric diseases.

## Highlights

► Abnormal neurodevelopment leads to fragmented NREM sleep in the MAM-E17 model ► Delta wave and spindle abnormalities match patterns of altered parvalbumin expression ► Spindle phase-locked units in frontal cortex fire during CA1 ripples in normal NREM ► Mistimed limbic-cortical oscillations during fragmented NREM may impair cognition

## Teaser

A restless pillow makes a ruffled mind? Investigating sleep architecture and associated neural network activity in an animal model of schizophrenia, Phillips et al. propose a new sleep-dependent mechanism for cognitive deficits in neuropsychiatric disease.

## Introduction

Structured neuronal activity spanning subcortical and cortical regions supports the integration and organization of recently learned information into stable, consolidated memory during sleep (see Diekelmann and Born, 2010). The extent to which distinct sleep stages and neurophysiological features differentially contribute to dissociable mnemonic processes remains unclear, but converging evidence indicates that cortical slow-waves, thalamocortical sleep spindles and hippocampal ripples during non-REM (NREM) sleep act in concert to preferentially support memory consolidation.

Increased numbers of slow-waves, spindles and ripples correlate with overnight improvements in declarative memories in humans and spatial memory in rodents (Diekelmann and Born, 2010), consistent with roles in processing hippocampal memory traces during NREM sleep. Reactivation of hippocampal ensemble firing occurs preferentially during CA1 sharp wave-ripples (O’Neill et al., 2010), which are in turn temporally correlated with thalamocortical sleep spindles (Siapas and Wilson, 1998; Sirota et al., 2003; Mölle et al., 2006); spindles themselves are phase locked to slow-waves and also associated with ensemble reactivation (Johnson et al., 2010). These temporal interrelationships may orchestrate the induction of synaptic plasticity during sleep by aligning replay of ensemble activity during ripples and spindles with periods of high cortical excitability during slow-wave “up states” (Diekelmann and Born, 2010). However, circuit mechanisms of ripple-spindle coordination and their dependence on global sleep architecture have not been directly demonstrated.

Given the interdependencies between neural activity during sleep and waking behavior, it is clear that sleep disruption may cause and/or exacerbate cognitive symptoms in diseases including schizophrenia, depression, Parkinson’s, Alzheimer’s, and Huntington’s disease (Wulff et al., 2010). In particular, schizophrenia-associated deficits in attention and memory processing may be attributed to aberrant sleep-related consolidation mechanisms (Manoach and Stickgold, 2009) and therefore be reflected by altered neural activity during sleep. Reductions in the number and power of slow-waves (Keshavan et al., 1998; Göder et al., 2006) and reductions in sleep spindle density (Ferrarelli et al., 2010; Manoach et al., 2010; Keshavan et al., 2011) correlate with either baseline cognitive deficits (Göder et al., 2006) or deficits in overnight memory recall (Göder et al., 2008; Manoach et al., 2010; Wamsley et al., 2012) in schizophrenia. These sleep abnormalities therefore constitute important targets for novel therapeutic intervention.

The MAM-E17 rat neurodevelopmental model of schizophrenia (Moore et al., 2006) employs administration of a mitotoxin MAM (methylazoxymethanol-acetate) to pregnant rats to induce a neurodevelopmental disruption, selectively targeting limbic-cortical circuits by timing embryonic day 17 MAM injections to coincide with hippocampal and prefrontal cortical embryogenesis (Lodge and Grace, 2009). Although no single intervention can model all aspects of schizophrenia in a rodent, the MAM-E17 model is therefore particularly useful in studying limbic-cortical dysfunction in neurodevelopmental disorders.

MAM-E17 exposed rats show cognitive changes reminiscent of those seen in schizophrenia, including impairments in spatial working memory (Gourevitch et al., 2004), attentional set-shifting (Featherstone et al., 2007), and reversal learning (Moore et al., 2006). MAM-E17 exposed rats also harbor glutamatergic dysfunction (Hradetzky et al., 2012), interneuronal dysfunction reflected by regionally restricted reductions in parvalbumin expression (Phillips et al., 2012) and reductions in the cortical up-state down-state transitions that underlie sleep slow oscillations (Moore et al., 2006).

Here, we use the MAM-E17 model to demonstrate the inter-dependence between sleep architecture, neocortical slow-wave propagation, and ripple-spindle coordination during NREM sleep, showing that neurodevelopmental disruption can lead to impaired hippocampal-prefrontal cortical network consolidation mechanisms.

## Results

### Fragmented NREM Sleep in E17 MAM Rats

Data from 14 SHAM and 13 MAM animals implanted with intracranial EEG electrodes over anterior motor cortex and posterior visual cortex (see Figure S1 available online) are presented here. Following a recovery period of 3 weeks, EEG, body temperature, locomotor activity, and food and water intake were recorded continuously for a period of 144 hr; results are taken from the final 48 hr of recording.

MAM-E17 rats exhibited robust circadian rhythms in all parameters measured, none of which differed significantly from controls (Figure S1). However, MAM-E17 animals did show a reduction in total NREM sleep (see Table S1 and Figure S1); this reduction was largest during the first 6 hr of the light phase (CT0–CT5) when controls slept the most (53.4% ± 1.4% NREM per hr), but remained significant during the second 6 hr period of the light phase (CT6–CT11) and the second 6 hr period of the dark phase (CT18–CT23). In contrast, there was no significant reduction in REM sleep. Time spent in each vigilance state and two-way-ANOVA results are presented in Table S1.

**Figure 1:**
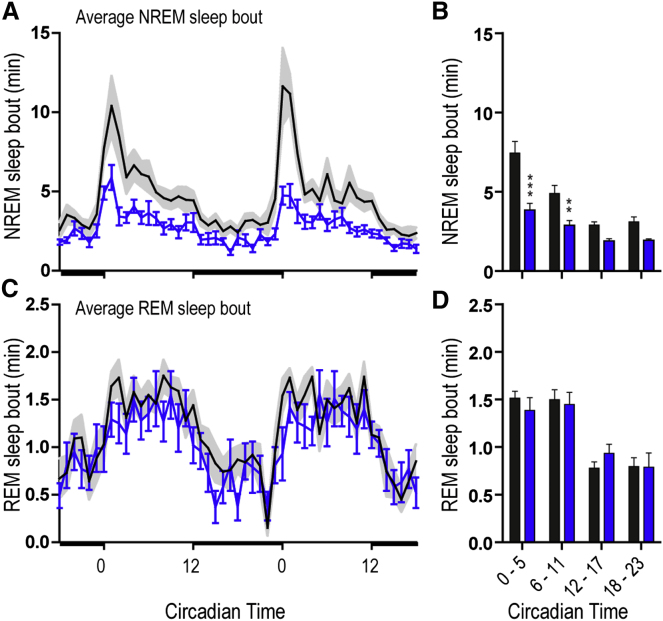
Fragmented NREM Sleep in E17-MAM Exposed Rats (A) Time course of average NREM sleep bout length (mean ± SEM) over a 48 hr recording period. Light/dark bars along the x axis indicate lights on/off. Note the longer sleep bouts at the beginning of the lights on (CT0-2) in SHAM (gray) which are reduced in the MAM rats (blue). (B) NREM sleep bout length over a 24 hr period averaged into 6 hr bins. (C and D) Equivalent time course and analysis of average REM bout lengths over the same period. Asterisks mark differences between SHAM (n = 14) and MAM (n = 13) groups (Bonferroni corrected t test ^∗∗∗^p < 0.001, ^∗∗^p < 0.01). See also Figure S1.

Sleep efficiency (time asleep/time spent in bed) in schizophrenia patients tends to decrease due to increased awakenings during the night (sleep fragmentation; see meta-analysis in Chouinard et al., 2004). Since rats have a polyphasic sleep cycle, we used sleep bout length as a measure of this sleep fragmentation (Figure 1). In controls, the first 1–2 hr of the light phase (CT0–CT1) were associated with the longest sleep bouts (10.4 ± 1.8 min); MAM animals had a marked 48% reduction in NREM sleep bout length, particularly between CT0–CT1 (Figures 1A and 1B). The average length of the longest REM bout was similar in both SHAM and MAM animals (Figures 1C and 1D).

Since sleep abnormalities in MAM-E17 rats were consistently restricted to NREM stages, we analyzed the neurophysiological features of NREM sleep in greater detail.

### NREM Delta Wave and Spindle Abnormalities in E17 MAM Rats

**Figure 2:**
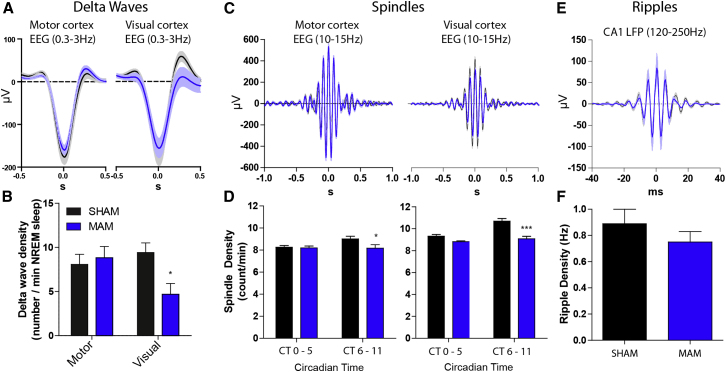
Selective Delta Wave and Spindle Abnormalities during NREM Sleep in E17-MAM Rats (A) Event triggered averages of raw EEG centered on the negative peaks of the delta waves from motor (left) and visual cortex (right) in SHAM (n = 14, black) and MAM (n = 13, blue) rats. (B) Delta wave densities from the motor and visual cortical EEG electrodes in SHAM and MAM animals. Delta wave density is significantly reduced over the visual cortex in the MAM animals (^∗^p < 0.05). Data are taken from the first 2 hr of the animals’ sleep period when slow-wave power is highest. (C and D) Average spindle waveforms (C) and densities (D) recorded over motor (left) and visual (right) cortex showing larger reduction of spindle density at posterior (^∗∗∗^p < 0.001) relative to anterior (^∗^p < 0.05) sites in MAM animals. (E and F) CA1 ripple waveforms and densities appear normal in MAM animals. All data shown as mean ± SEM. See also Figures S1 and S2.

Individual delta waves (0.3–3Hz) were detected in EEG at both anterior (motor cortex) and posterior (visual cortex) recording sites across the entire light phase (n = 8; Figures 2A, 2B, and S2). MAM and SHAM EEG showed similar delta wave densities and amplitudes over motor cortex. In contrast, we found a small change in the amplitude (MAM = 156.2 ± 21.8, SHAM = 172.6 ± 23.8μV) and a 50% reduction in the density of NREM delta waves over the visual cortex in MAM animals (MAM = 4.7 ± 1.2, SHAM = 9.4 ± 1.1 waves/min; p < 0.05; Figure 2B). Accordingly, spectral analysis of NREM sleep EEG showed a clear reduction in delta power (Figure S1).

Thalamocortical spindles (10–15 Hz) were most prominent on anterior EEG electrodes (Figure 2C). There was a slight but significant reduction in spindle density during NREM sleep in MAM animals recorded over both visual (−15.1%, p < 0.001) and motor cortices (−9.2%, p < 0.05; Figures 2D and S2), particularly in the second half of the sleep period. The reduction in spindle density recorded over motor cortex was not associated with any change in spindle properties (Figure 2C, left panel). There was, however, a reduction in the amplitude of spindles recorded over visual cortex of MAM animals (−30%, p < 0.01; Figure 2C), although mean frequency and length remained similar to controls (p > 0.05; Figure S2).

Ripple oscillations (120–250 Hz) in the hippocampus are a prominent feature of NREM sleep not evident in surface EEG. We performed unilateral, dual-site medial prelimbic cortex (PrL) and dorsal CA1 tetrode recordings of local field potential (LFP) and multiple single neuron spike trains to monitor CA1 ripples and PrL spindles. Hippocampal ripple intrinsic frequencies (MAM = 182 ± 1, SHAM = 179 ± 3 Hz), peak amplitudes (121 ± 23 versus 119 ± 18 μV), lengths (36.6 ± 1.9 versus 37.3 ± 2.4 ms), and densities (0.89 ± 0.11 versus 0.75 ± 0.08 Hz) were normal in MAM animals (Figures 2E and S2), indicating that basic hippocampal circuitry of ripple generation was spared following E17 MAM exposure.

Altogether, mechanisms of delta wave and spindle generation in anterior/motor cortical areas appear largely intact in MAM-E17 exposed rats, as does the circuitry responsible for hippocampal ripples. In contrast, delta wave and spindle density at posterior/visual cortical sites is preferentially attenuated. Given the coupling between delta, spindle and ripple oscillations in rodents and humans (Siapas and Wilson, 1998; Clemens et al., 2007), we next sought to analyze temporal relationships between these network oscillations.

### Impaired Cortical Delta Wave Synchrony

In humans, delta waves originate more frequently in frontal regions and propagate through the cortex in an anteroposterior direction as traveling waves (Massimini et al., 2004). We therefore tested whether the reduced delta-wave density seen at posterior sites resulted from reduced anteroposterior slow-wave propagation in MAM-exposed rats.

**Figure 3:**
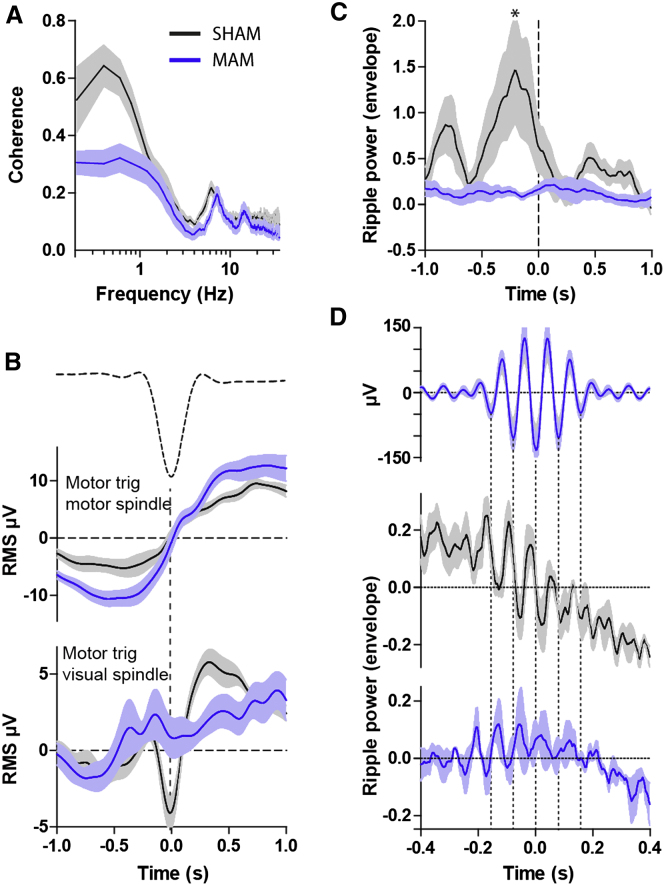
Delta Waves, Spindles, and Ripples Are Desynchronized in MAM Animals (A) Coherence between motor and visual cortical EEG during NREM sleep (bandwidth 0.2 Hz, 10 s moving window, 4 tapers) is significant in the 0.3–3 Hz frequency range in SHAM animals (black, n = 8, p < 0.05) and significantly reduced in the MAM animals (blue, n = 8, p < 0.01 versus SHAM), indicating impaired anterior-posterior delta wave coordination. (B) Motor delta wave (dotted line shows average delta waveform)-triggered spindle power shows similar patterns at anterior sites in SHAM and MAM animals (upper graph) but is severely disrupted at posterior cortical sites (lower graph) in MAM animals. Note the characteristic occurrence of spindles on the rising phase of delta waves in SHAM animals. (C) PrL spindle-triggered averaging of CA1 ripple power (from LFP, 10 ms bin width) shows that ripples consistently precede spindles in SHAM animals (black, n = 6) but that this timing relationship is abolished in MAM animals (blue, n = 5; Bonferroni corrected pairwise comparison ^∗^p < 0.05). (D) Alignment of average PrL spindle waveforms (upper graph) with ripple band RMS activity shows fine timescale, spindle-frequency modulation of CA1 ripples in SHAM animals (middle graph) and its attenuation in MAM animals (lower graph). All data shown as mean ± SEM. See also Figure S3.

We aligned the start times of first long NREM sleep bouts in the light phase and averaged the magnitude of Fourier coherence between motor and visual cortical electrodes across animals (Figure 3A). There was significant coherence (0.52 ± 0.14, p < 0.05) between the motor and visual cortical EEG electrodes in the 0.3–3Hz frequency range which was significantly reduced in the MAM animals (0.29 ± 0.11; p < 0.01 versus SHAM; Figure 3A). Cross-correlations centered on the visual delta waves (FigureS3) showed a leftward-shifted peak in controls, indicating that on average anterior, motor cortical delta waves precede posterior, visual cortical waves by ∼50 ms. This peak was significantly reduced in MAM animals (group × time interaction p < 0.0001), again indicating impaired propagation and long-range coordination of cortical delta waves in the MAM-E17 model.

### Impaired Phase Locking of Spindles to Delta Waves

We next investigated whether the timing of thalamocortical spindles relative to delta waves was altered in MAM rats. Event triggered averages of 10–15 Hz band-pass-filtered EEG time-locked to the peak of delta waves in motor cortex show a clear relationship between slow oscillations and spindle activity in SHAM animals (Figure 3B): 400–600 ms before the peak negativity of the delta-waves, spindle activity decreased to an absolute minimum (−6.3 ± 1.5 μV), followed by a strong spindle rebound covering the entire subsequent positive-going phase of the delta-waves to +4.5 ± 0.5 μV; p < 0.0001 pre versus post). This depression and subsequent rebound was even more pronounced over the visual electrode (−1.8 ± 2.4 μV versus +5.4 ± 0.7 μV; p < 0.0001; Figure 3B, lower panel). In MAM animals, the motor cortical reduction and rebound in spindle activity around the delta-wave remained intact (−5.4 ± 1.8 μV versus +4.7 ± 2.4 μV, p < 0.0001; Figure 3B), whereas visual cortical spindle activity was not influenced by the timing of motor cortical delta-waves (+1.4 ± 1.2 μV versus +1.6 ± 0.78 μV, p > 0.05). Again, these results indicate preferential disruption of network activity during NREM sleep at posterior cortical sites, likely as a consequence of the impaired anteroposterior delta wave coordination in the MAM model.

### Disrupted Ripple-Spindle Synchronization

We next used CA1-PrL tetrode recordings to identify the temporal relationship between hippocampal ripples and PrL spindles. First, we calculated power in the hippocampal ripple band and cortical spindle band using a 500 ms sliding average window and performed cross-correlation analysis on the resulting power changes over time. This revealed a robust temporal relationship between hippocampal ripple and PrL spindle power in SHAM animals: in 5 out of 6 SHAM animals, ripple power consistently peaked within 500 ms prior to spindle power, whereas in MAM animals the magnitude of the correlation and the temporal coupling to the PrL spindle was greatly reduced (Figure S3). Similarly, PrL spindle-triggered averages of CA1-LFP filtered at ripple frequency (120–250 Hz) showed twin peaks of ripple power in 5 of the 6 SHAM animals: the largest main peak occurred 244 ± 67 ms prior to spindles, while a second smaller peak was apparent at 849 ± 40 ms (Figure 3C). In stark contrast, no peaks or troughs were observed in the MAM animals, indicating a profound decoupling of hippocampal and cortical networks during NREM (p < 0.01).

To characterize finer timescales of ripple-spindle coupling, grand averages centered on individual spindle maxima were computed from hippocampal CA1 LFP filtered at ripple frequency (Figure 3D). In addition to confirming that ripples tend to precede spindles in normal rats, this analysis revealed a strong modulation of CA1 ripple power by PrL spindle oscillations in SHAM animals reminiscent of the ripple-spindle relationship reported in humans (Clemens et al., 2011). Spindle modulation of ripple power was completely lacking in some MAM-exposed animals, and on average grossly reduced in amplitude compared to SHAM animals (Figure 3D).

E17-MAM exposure therefore spares the intrinsic properties of ripples and spindles but leads to selective decoupling of ripple-spindle coordination likely to disrupt systems consolidation mechanisms.

### PrL Spindle Phase Locking Predicts PrL-CA1 Unit Cross-correlation

**Figure 4:**
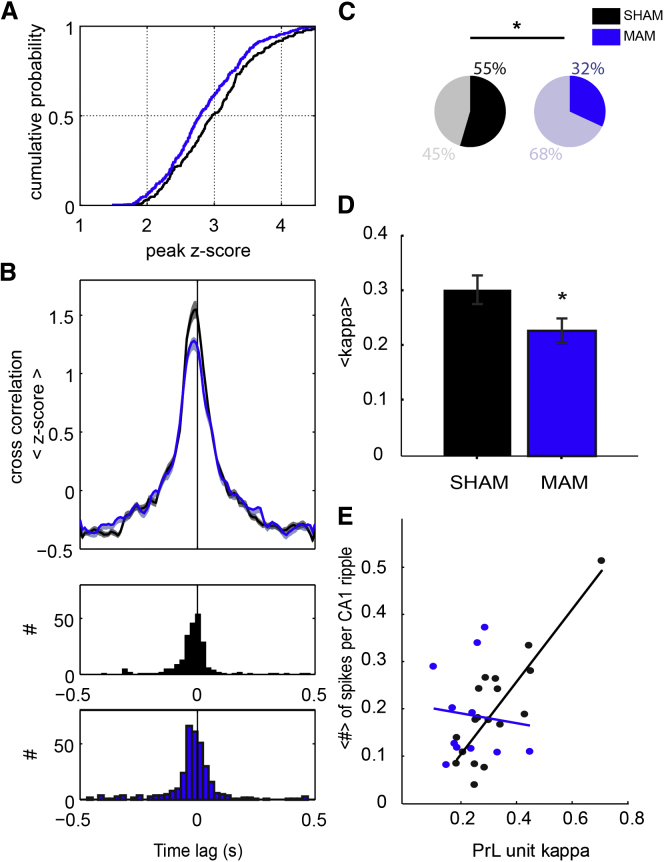
Attenuated CA1-PrL Unit Cross-correlations and Spindle Phase Locking in E17-MAM Rats (A) Left-shifted cumulative distribution function of peak cross-correlation values between CA1 and PrL pyramidal cell spike times during ripples in MAM animals (blue) relative to SHAM controls (black). (B) Mean ± SEM normalized cross-correlations between CA1 (time lag = 0 s) and PrL units for SHAM (black, n = 4, n = 232) and MAM (blue, n = 4, n = 332) animals showing a significantly reduced short-latency peak in MAM animals (p < 0.05, Kolmogorov-Smirnov test). Lower panels plot distributions of CA1-PrL cross-correlation peak times; the left-shifted peak indicates that PrL units tend to fire before CA1 units during ripples in MAM-animals. (C) The percentage of significantly spindle phase-locked putative pyramidal cells in PrL is higher in SHAM (55%, total n units = 38) than MAM rats (32%, total n units = 46). Fisher’s exact test for binomial distributions indicates a significant difference in the percentages (^∗^p < 0.05). (D) Mean ± SEM circular concentration coefficient (kappa) values for all significantly phase locked PrL units in SHAM (black) and MAM (blue) animals (^∗^p < 0.05, t test). (E) Regression analysis showing that the extent of PrL spindle phase locking (kappa) correlates with the proportion of spikes fired during CA1 ripples for PrL putative pyramidal cells in SHAM animals (black, R^2^ = 0.72, p < 0.0001) but not MAM animals (blue, R^2^ = 0.1, p > 0.5). This correlation holds without inclusion of the outlying SHAM unit with kappa = 0.7. See also Figure S4.

We next tested whether the spike timing of extracellularly recorded multiple single units in PrL and CA1—particularly in relation to ongoing LFP oscillations—was affected by MAM exposure (Wierzynski et al., 2009). Although the number of spikes fired during ripples (see Figure S4) and spindles (see below) appeared normal in MAM animals, cross-correlations between PrL and CA1 spikes occurring within 250 ms time windows around ripple maxima were significantly reduced in MAM animals (p < 0.05, Kolmogorov-Smirnov test; Figures 4A and 4B; see Figure S4). The relative timing of CA1-PrL spiking also appeared shifted in MAM animals, in which there was a greater tendency for PrL spikes to precede CA1 spikes (Figure 4B).

Putative PrL pyramidal cell units were classified according to spike width and firing rates (see Experimental Procedures and Figure S4) and their spiking relative to local spindle oscillations examined (see example in Figure S4). In SHAM rats, 55% of units showed firing significantly phase locked to PrL spindles (p < 0.05, Rayleigh test of uniformity); this was higher than the proportion of phase-locked units in MAM animals (32%; p < 0.05 versus SHAM, Fisher’s exact test; Figure 4C) and could not be explained by differing spindle-associated spike numbers (SHAM 535 ± 141 spikes, MAM 568. ± 110 spikes, p = 0.86). Considering only significantly phase-locked units from SHAM and MAM animals, mean circular concentration coefficients of phase-locking were lower in MAM animals (p < 0.05; Figure 4D), reflecting less reliable phase locking of putative pyramidal cells to ongoing spindle oscillations in MAM animals.

Combining the two unit analyses described above we show for the first time in normal animals that PrL units with the most robust spindle phase locking fire a greater proportion of their spikes during hippocampal ripples than less spindle phase-locked units (see linear regression in Figure 4E). This relationship did not hold in MAM animals: even significantly spindle phase-locked PrL units did not show any tendency to be more active during CA1 ripples. This is consistent with the reduced ripple-spindle coordination and CA1-PrL decoupling during NREM sleep in MAM rats and details novel, sleep-dependent network and single cell electrophysiological mechanisms likely to contribute to cognitive deficits in a psychiatric disease model.

## Discussion

We show that impaired anteroposterior propagation of cortical delta waves in the MAM-E17 model is associated with mistiming of spindle oscillations during NREM sleep; hippocampal ripple events consequently fail to coordinate with spindles in directly connected neocortical regions, including PrL. The decoupling of CA1-PrL networks during NREM sleep in the MAM-E17 model is likely to reflect disrupted sleep-dependent memory consolidation mechanisms and to model sleep abnormalities that contribute to cognitive dysfunction in diseases like schizophrenia.

Alongside sleep fragmentation, a wide range of sleep abnormalities have been reported in schizophrenia (Keshavan et al., 1990; Manoach and Stickgold, 2009), including increased sleep latency, increased wake time after sleep onset, and diminished sleep efficiency (Benca et al., 1992; Chouinard et al., 2004). A number of studies confirm reductions in NREM, slow-wave sleep (SWS or N3) that correlate with measures of cognitive disorganization, impaired attention and disrupted declarative and procedural memory, hence impaired SWS is consistently linked to cognitive symptoms (Göder et al., 2006; Yang and Winkelman, 2006; Sarkar et al., 2010). Conversely, schizophrenic patients with mild cognitive symptoms do not show robust SWS deficits (Ferrarelli et al., 2010).

Few animal models have been examined for sleep abnormalities and the impact of sleep disruption on the circuit basis of cognition has remained largely unexplored. MAM-E17 exposed rats model a wide range of neuroanatomical abnormalities associated with schizophrenia (Lodge and Grace, 2009), including reduced frontal cortical thickness, increased ventricle volume (Moore et al., 2006), and a loss of prefrontal cortical and ventral hippocampal parvalbumin-expressing (PV+) interneurons (Lodge et al., 2009). Here, we show that MAM-E17 rats also show a reduced amount of NREM sleep that occurs in shorter bouts than in normal animals, but no change in the occurrence of REM sleep. This fragmented sleep architecture bears a striking similarity to that seen in at least a subset of schizophrenia patients (Wulff et al., 2010) and has been associated with increased ventricle size—also evident in E17-MAM rats—in humans (van Kammen et al., 1988). The MAM-E17 model thereby presents a unique opportunity to demonstrate links between neuropathology, sleep architecture and sleep neurophysiology.

Interplay among spontaneous synaptic inputs, intrinsic neural properties, and coupled thalamocortical network oscillations generates EEG power in the 0.3–3 Hz frequency range (Crunelli and Hughes, 2010). The reduced delta power during NREM sleep in MAM-E17 animals could therefore arise through cortical dysfunction, altered thalamocortical input, or both. Individual delta waves of normal amplitude could still be detected in MAM-E17 rats; their numbers during NREM sleep were maintained in motor cortex EEG but significantly reduced in EEG recorded over visual cortex (Figure 2). These data indicate that the basic circuitry of delta wave generation is intact in the MAM-E17 model. However, the reduced proportion of prelimbic cortical pyramidal cells exhibiting up-down state fluctuations in anesthetized MAM-E17 rats (Moore et al., 2006) might reflect an impaired ability of cortical networks to maintain, synchronize or propagate delta waves through larger areas of cortical tissue. Indeed, loss of coherence in the delta band and a significantly reduced cross-correlation between individual delta waves in MAM-E17 animals (Figure 3) shows that synchronization between cortical sites—which is increased following learning in humans (Mölle et al., 2004)—is disrupted.

Loss or dysfunction of cortical PV^+^ interneurons, which play pivotal roles in timing pyramidal cell activity but are reduced in both postmortem tissue from patients (Lewis et al., 2005) and in the MAM-E17 model (Lodge et al., 2009; Phillips et al., 2012), may impair the coordinated, sequential activation of intracortical circuits that presumably underlies slow-wave propagation. As in schizophrenia (Ferrarelli et al., 2010), MAM-17 rats also show a small reduction in sleep spindle density, which may again reflect PV^+^ dysfunction given the prevalence of PV^+^ cells in spindle-initiating reticular thalamus, plus the participation of PV^+^ cortical basket cells in spindle oscillations (Hartwich et al., 2009). Indeed, thalamic abnormalities are an increasingly recognized feature of schizophrenia (Adriano et al., 2010).

Our control data confirm that the onset of thalamocortical spindles precedes an increase in delta power, and that maximum spindle power coincides with the up-state of cortical slow oscillations (Mölle et al., 2006). This temporal relationship between spindles and delta waves is intact around the anterior initiation site in MAM-E17 animals and the intrinsic properties of their spindles do not differ from SHAM controls, indicating that some thalamocortical circuit function is maintained. However, the spindle-delta power correlation is strongly diminished over MAM-E17 posterior cortical regions, presumably as a consequence of impaired delta wave propagation. This means that posterior cortical spindles are mistimed relative to pyramidal cell depolarization states in MAM-E17 animals, potentially attenuating the functional impact of spindle-associated firing patterns. Further evidence for mistiming of spindle initiation in the MAM-17 model comes with the most striking result of the current study, namely the loss of synchronization between hippocampal ripples and cortical spindles (Figure 3).

The temporal coupling of hippocampal ripples and cortical spindles during NREM has been demonstrated in both rats and humans (Siapas and Wilson, 1998; Sirota et al., 2003; Mölle et al., 2006; Clemens et al., 2007), and recent human studies suggest that delta waves coordinate frontal and temporal cortical activity during sleep (Nir et al., 2011). This may arise via cortical input modulating ripple initiation (Sirota et al., 2003; Isomura et al., 2006; Mölle et al., 2006). Here, we extend these previous results, showing spindle phase locking of hippocampal ripple power similar to that reported in humans (Clemens et al., 2011) in SHAM animals (Figure 3). Embedded slow-wave, spindle, and ripple oscillations therefore coordinate the rhythmic firing of pyramidal cells in cortex and CA1, providing windows of opportunity for cross-structural synaptic plasticity. Indeed, oscillatory activity in both hippocampus and neocortex during NREM sleep is associated with selective reactivation of activity sequences seen during previous behaviors (Peyrache et al., 2009; O’Neill et al., 2010). The initiation of this replay through cortical delta wave-modulated input may mark the beginning of a looped circuit interaction, whereby cortical delta waves initiate hippocampal reactivation during ripples, which in turn triggers cortical reactivation during spindles (Marshall and Born, 2007).

The lack of coupling between hippocampal ripples and cortical spindles in MAM-17 rats demonstrates the crucial role of synchronized cortical slow-waves in organizing the dialog between cortex and hippocampus by providing a temporal framework for faster oscillations. Disrupting this dialog presumably constitutes the neurophysiological mechanism for behavioral deficits in long term learning and memory described in the MAM E17 model (Flagstad et al., 2004; Gourevitch et al., 2004; Moore et al., 2006), and may contribute to cognitive deficits in other models of sleep fragmentation (Tartar et al., 2006).

Our study serves to emphasize that disrupted thalamic-cortical-limbic network activity during sleep must therefore be considered alongside waking activity as a therapeutic target in schizophrenia and related diseases. Since active entrainment of slow-waves through transcranial stimulation enhances both spindle density and declarative memory in humans (Marshall et al., 2006) one intuitive possibility would be to use transcranial stimulation as a possible therapy for relieving cognitive and sleep deficits found in patients. The MAM-E17 model provides a unique opportunity to study the detailed cellular, synaptic and network mechanisms that underpin such novel therapeutic approaches.

## Experimental Procedures

All procedures were carried out in accordance with the UK Animals Scientific Procedures Act (1986) and University of Bristol and Lilly UK ethical review. Sprague-Dawley dams were obtained from Charles River (UK) on day 12 of gestation and injected on E17 with saline or MAM (22 mg/kg i.p.; Midwest Research Institute, Missouri). Fifteen saline-injected and 15 MAM-injected dams produced 51 SHAM and 49 MAM pups. No more than two animals used were derived from a single litter.

In brief, 70–80 day old rats were prepared for either EEG recording (cranial implant of five stainless steel screws: 2× motor cortex +3.9 mm AP, ± 2.0 mm ML, 2× visual cortex −6.4mm AP, ±5.5 mm ML from bregma) or LFP/multiunit recordings (arrays of adjustable tetrode recording electrodes targeted to the PrL +3.2 mm, +0.6 mm from bregma and ipsilateral dorsal CA1 −3.6 mm, +2.2 mm). For EEG recordings, sleep/wake parameters were monitored using SCORE-2004, an updated version of a real-time sleep/wake monitoring system (Van Gelder et al., 1991), with bout length defined as continual episodes of NREM/REM not interrupted by two or more consecutive 10 s epochs of wake. Tetrode recordings were made immediately following exploration of a linear maze in order to ensure slow-wave and spindle-rich sleep and putative pyramidal cell spike times indentified based on clustering, waveform and firing-rate parameters. Delta wave, spindle, and ripple events were detected based on standard filtering and thresholding algorithms validated by independent visual scoring. See Supplemental Experimental Procedures for detail.

Unless otherwise stated data are expressed as mean ± SEM t tests or repeated-measure ANOVAs were used to test for significant differences between MAM and SHAM (identified in Results). Significant ANOVA effects were followed by Bonferroni t test to correct for multiple comparisons. Normality was checked using D’Agostino & Pearson omnibus normality test (Graphpad software).

## Supplemental Information

Document S1. Figures S1–S4, Table S1, and Supplemental Experimental Procedures
