# Supplementary material for: Decoupling of Sleep-Dependent Cortical and Hippocampal Interactions in a Neurodevelopmental Model of Schizophrenia
Source: Neuron. 2012 Nov 8;76(3):526–33. doi: 10.1016/j.neuron.2012.09.016 (PMC3898840; doi:10.1016/j.neuron.2012.09.016)
Supplement: Document S1. Figures S1–S4, Table S1, and Supplemental Experimental Procedures [file mmc1.pdf]

Neuron, Volume *76*

Supplemental Information

Decoupling of Sleep-Dependent Cortical and Hippocampal Interactions in a Neurodevelopmental Model of Schizophrenia

Keith G. Phillips, Ullrich Bartsch, Andrew P. McCarthy, Dale M. Edgar, Mark D. Tricklebank, Keith A. Wafford, and Matt W. Jones

Supplemental Items Inventory

These supplementary items are essential in order to confirm further methodological and analytical details and demonstrate the accuracy and selectivity of effects shown in the main figures.

Figure S1

Figure S1 shows further details of sleep architecture and EEG recordings described in Figures 1 and 2. Panels A-D show variables measured during home cage EEG recording to support a NREM specific deficit in MAM treated animals. Panel E shows electrode locations and example EEG traces. Panels F-I show example spectrograms and delta power increase from the start of a sleep bout to strengthen the notion of specific delta power decrease during NREM sleep in MAM animals.

Figure S2

Figure S2 shows detailed properties of NREM sleep oscillatory events quantified in Figure 2. Panel A shows detailed slow wave properties (EEG), panel B shows detailed spindle properties (EEG) and panel C shows ripple properties (LFP). Panel D shows the peri-event time histogram of hippocampal single units in relation to the maximum amplitude of detected ripples, demonstrating normal ripple-related pyramidal cell firing in CA1 of MAM-exposed animals.

Figure S3

Figure S3 further details temporal relationships between network oscillations during NREM sleep shown in Figure 3. Panels depict averaged slow wave coherence (A1) and cross-correlations between detected slow wave events (A2). Panels B1-3 show example and average cross-correlograms between ripple and spindle power to confirm PrL-CA1 decoupling during NREM sleep.

Figure S4

Figure S4 supports the results of the unit analysis presented in Figure 4. Panels A1-A4 show example histological confirmation of tetrode positions in SHAM and MAM animals. Panel B shows an example recording of simultaneous LFP and unit recording in PrL and CA1 during REM sleep to illustrate the temporal relationship of slow waves, ripples and spindles in SHAM animals. Panels C1-2 show the distribution of spike widths in relation to firing rate of the isolated units and the limits applied for the analysis displayed in Figure 4. Panel D1-2 shows an example cross-correlograms for all spikes during NREM and spikes limited to a 250 ms window around ripple peak time.

Table S1

Table S1 shows detailed statistics on sleep architecture comparison between SHAM and MAM animals.

Supplemental Experimental Procedures

The Supplemental Experimental Procedures add further detail to the Experimental Procedures section.

Supplemental Figure S1

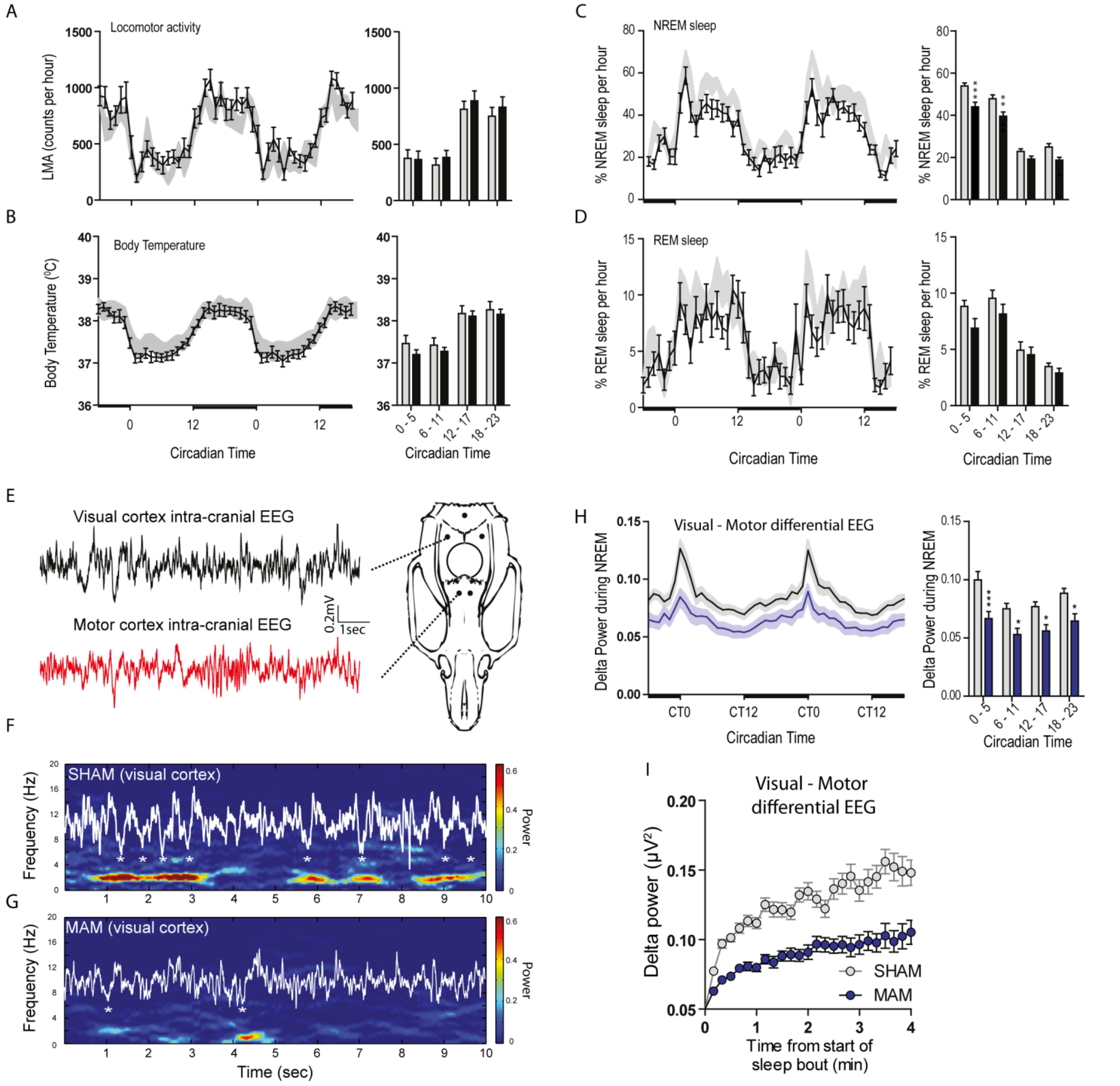

Time course of (A) locomotor activity, (B) body temperature and (C**)** NREM sleep expressed as % NREM per hour over a 48h undisturbed period; note the peaks in NREM sleep at the beginning of the lights on phase (CT0-2). Repeated measures ANOVA indicates a significant group ($F_{(1,25)}$ =16.4; p < 0.001) and time effect ($F_{(3,25)}$=261.4 p < 0.0001) (D) REM sleep, expressed as % REM sleep per h over a 48h period. Bars indicate data averaged into 6h bins. (E) Schematic diagram to indicate location of EEG recordings with example EEG taken from a SHAM animal. (F,G) 10s example visual cortical EEG trace, overlaid on the corresponding spectrogram taken 30s before the end of the first long sleep bout in the light phase (CT0). (F) Representative example taken from a SHAM animal, (G) is taken from the equivalent time point in the sleep bout from a MAM animal.* denote delta waves. (H) Time course of mean delta power (0.3-3Hz) ± SEM in NREM epochs from visual-motor differential EEG, expressed as μV^2 and averaged into hourly bins, over a 48h period. Black line = SHAM, blue line = MAM treated rats; shaded area indicates SEM. Bars indicate delta power in NREM epochs over a 24 hour period averaged into 6 hourly bins. Repeated measures ANOVA shows a significant group ($F_{(1,25)}$=8.1; p < 0.01) and group x time interaction ($F_{(3,75)}$=4.5; p < 0.01). (I) Delta power (0.3-3Hz) over time from the start of aligned sleep bouts. SHAM animals have steep initial increases in delta power that continue to rise more slowly throughout the sleep bout. MAM animals have a significantly shallower gradient in the delta power rise (p < 0.001). Data plotted as mean ± S.E.M. with light/dark bars along the x-axis indicating

lights on/off. In all cases significant differences between the SHAM and MAM groups compared using a Bonferroni corrected t-test are indicated by an asterisk at the top of the plots (*** = p < 0.001, ** = p < 0.01, * = p < 0.05). Data were obtained from 14 SHAM and 13 MAM-treated rats.

Supplemental Figure S2

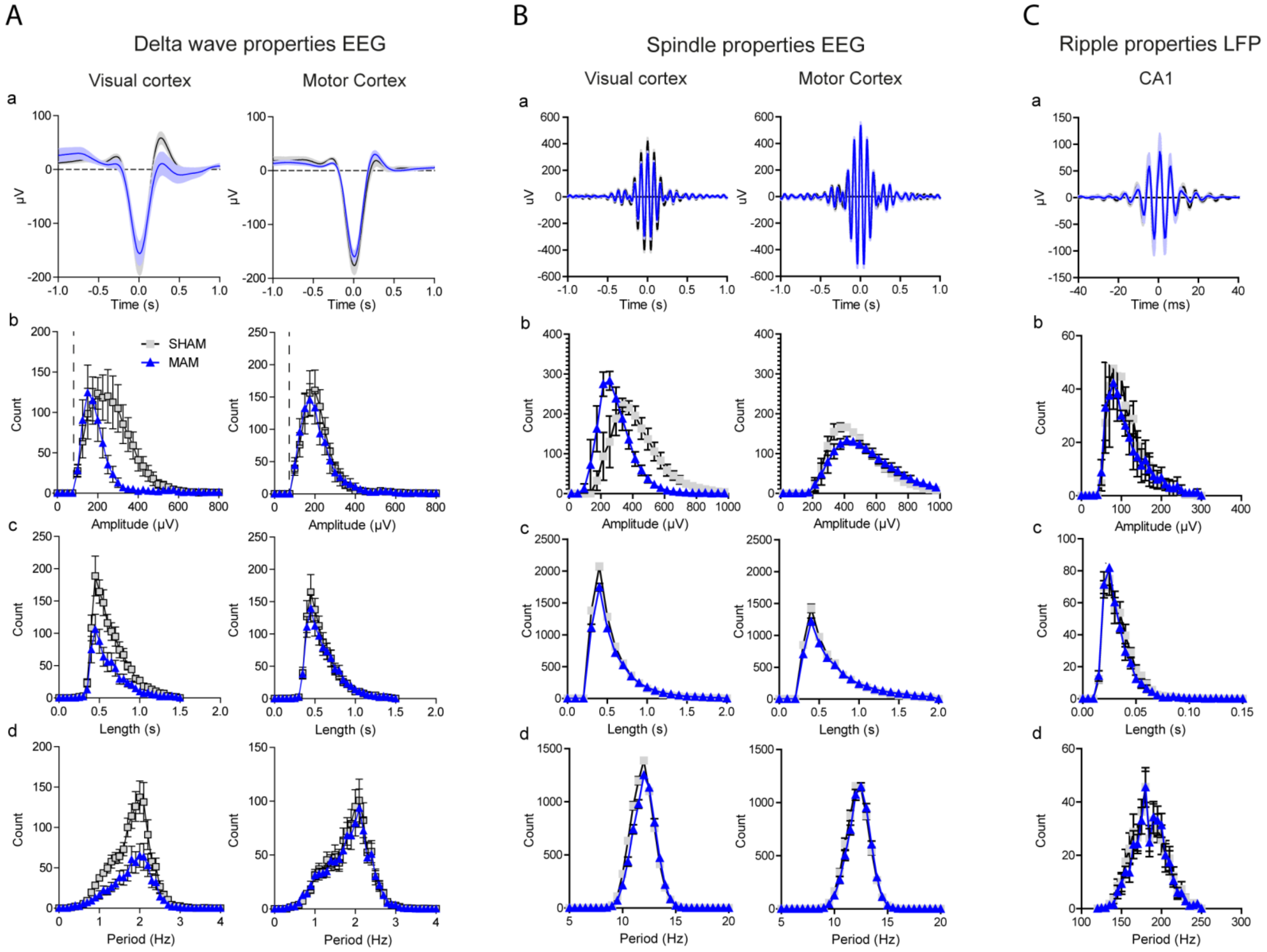

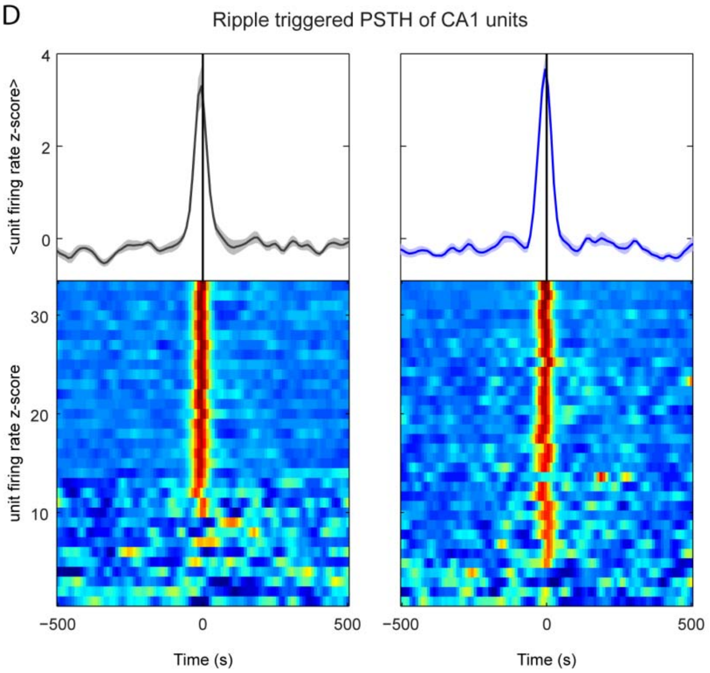

(A) Delta wave properties (A1) Grand averages of delta wave triggered EEG recorded over visual (left) and motor cortices (right) from SHAM (black line) and MAM animals (blue line n=8 per group). Average histograms of all delta waves detected from SHAM (black) and MAM (blue) showing the distribution of delta wave amplitudes (**A2**) lengths (**A3**) and frequencies (**A4**) between the two groups. Dotted lines in A2 indicate the threshold for the detection algorithm. **(B) Spindle properties** (**B1**) Grand averages of spindle triggered EEG recorded over visual (left) and motor cortices (right) from SHAM and MAM animals. Average histograms of all spindles detected between CT0-11 from MAM (blue) and SHAM (black) showing the distribution of spindle amplitudes (**B2**) lengths (**B3**) and frequencies (**B4**) between the two groups. (**C**) **Ripple properties** (**C1**) Grand averages of ripple triggered 140-240Hz filtered CA1 depth LFP recorded from SHAM (black line) and MAM (blue line) animals. Average histograms of all ripples detected from MAM (blue) and SHAM (black) showing the distribution of ripple amplitude (**C2**) length (**C3**) and frequency (**C4**) between the two groups. (**D**) Peri-event time histograms (PETH) showing similar firing of CA1 putative pyramidal cells during ripples in SHAM and MAM animals. Upper panels show mean, normalized PETHs centred on ripple maxima (Time lag = 0) for 33 SHAM (left, N=4) and 34 MAM (right, N=4) CA1 units included according to the firing rate criteria used by Wierzynski et al. (2009). Shaded area shows mean±sem. Lower panels show color-scaled PETHs for all individual units.

Supplemental Figure S3

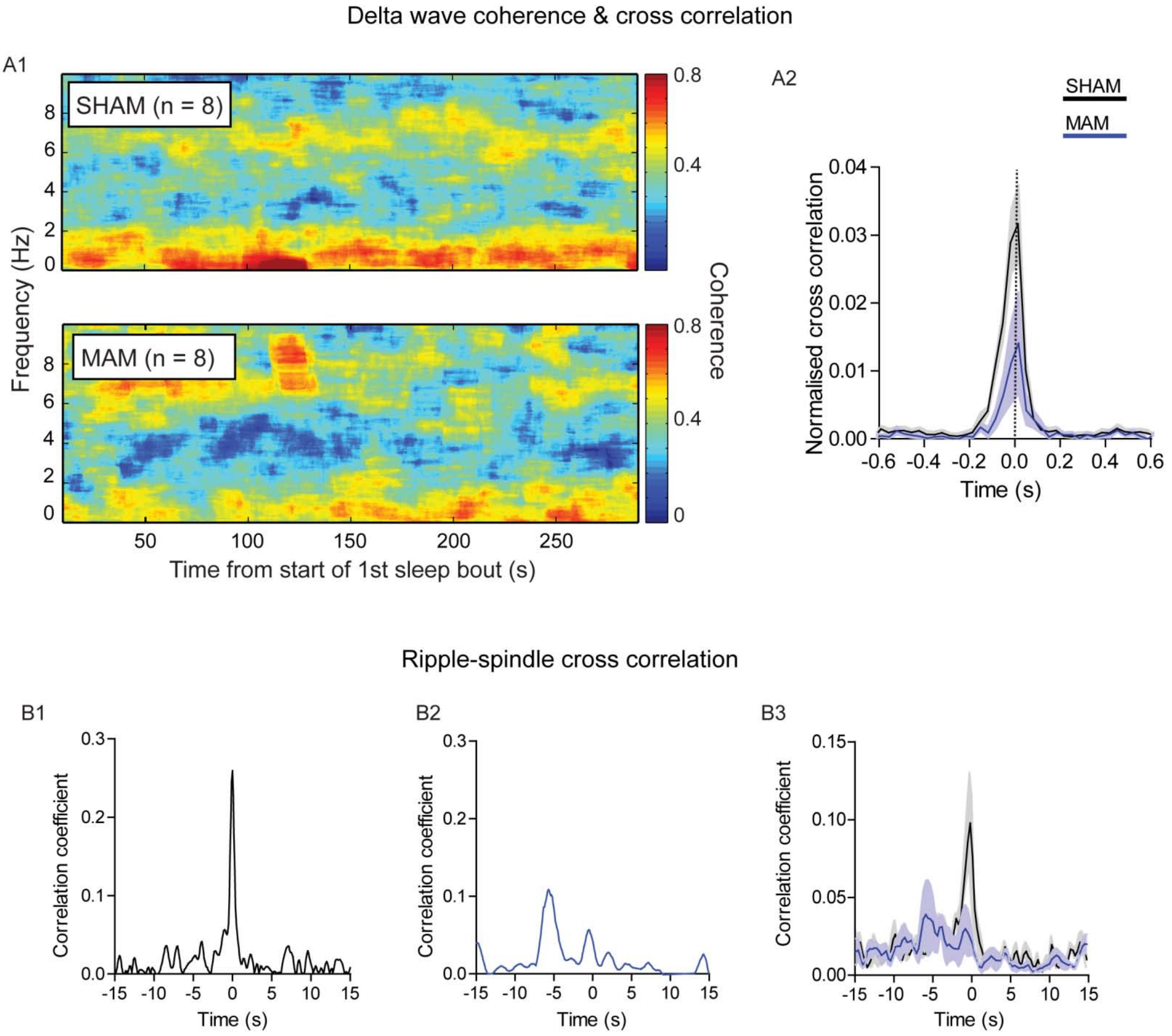

(**A1**) Average multi taper coherogram plot, showing coherence between motor and visual EEG recording sites during NREM sleep at different frequencies over time. Sleep bout onset times in the light phase were aligned across animals in SHAM (top) and MAM (bottom) to allow coherence within a sleep bout to be averaged. The bandwidth was set at 0.2Hz and a 10sec moving window was used. The SHAM animals show high levels of coherence in the 0.3-2Hz frequency range, which is reduced in the MAM animals. (**A2**) Motor visual slow-wave cross correlation referenced to visual cortical slow-waves. The peak in the cross correction is offset to the left indicating that on average anterior slow-waves precede slow-waves in the posterior, visual cortex. The synchrony of cortical slow-waves is reduced in the MAM animals as shown by the reduction in the cross correlation peak (group x time interaction $F_{(37,\ 444)}$=4.3; p < 0.0001). Data are normalized to the total number of slow-waves detected during the recording period. (**B**) Examples of a cross-correlogram of spindle power to ripple power in a single control SHAM (**B1**) and MAM animal (**B2**). The clear peak in the ripple spindle cross-correlogram at ~-300ms in the SHAM animal is clearly shifted and reduced in the MAM animal. (**B3**) Group data (SHAM n= 6, MAM n= 5) showing a significant reduction in the temporal correlation of ripple-spindle times in the MAM animals (blue line) compared to control SHAM animals (black line) (group x time interaction ($F_{(1,\ 59)}$=2.1; p < 0.001).

Supplemental Figure S4

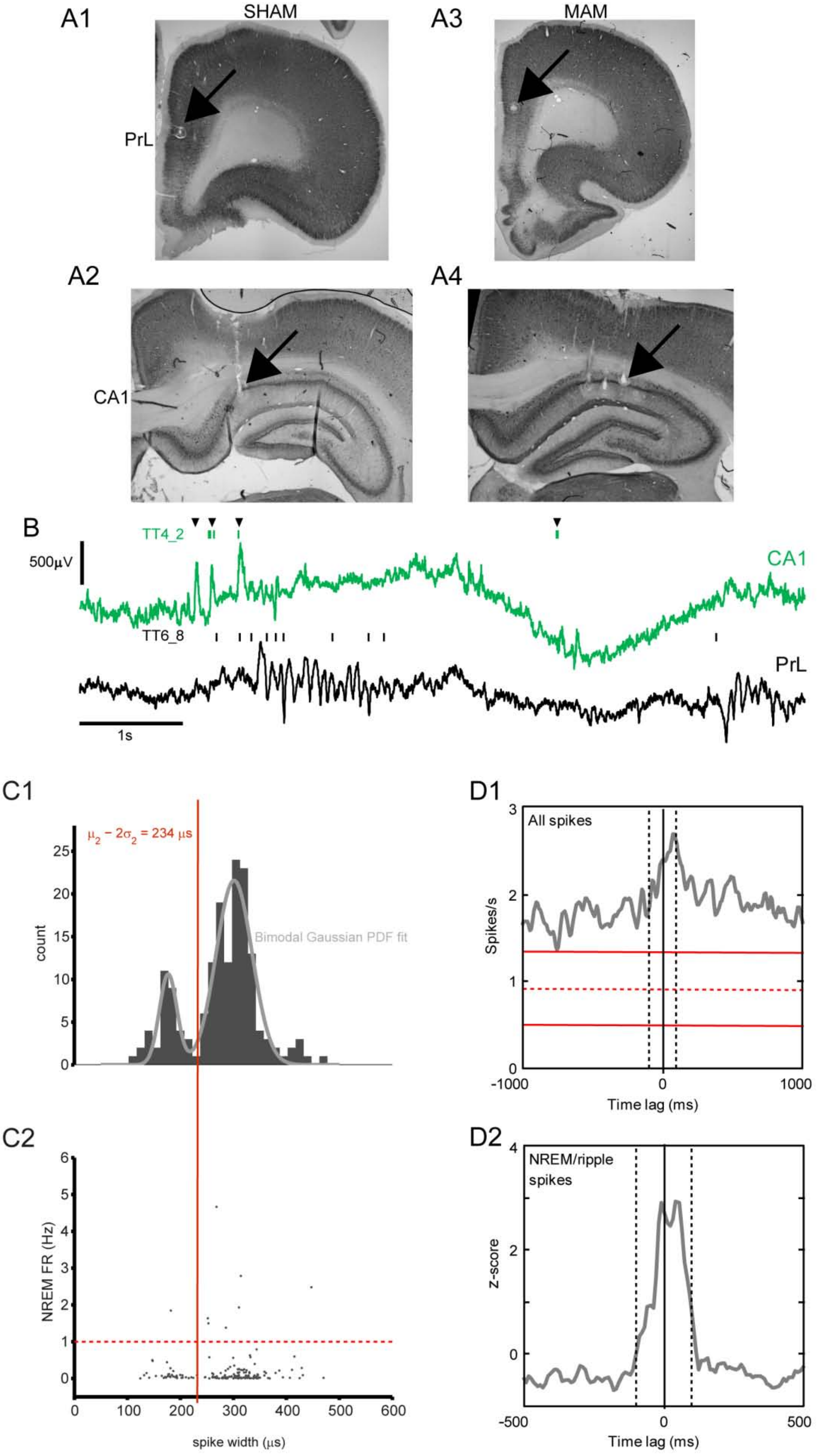

(**A**) Example histological verification of tetrode positions for both SHAM (A1, A2) and MAM (A3, A4) animals in the mPFC and dorsal CA1. Black arrows indicate post-mortem lesions. (**B**) CA1-PrL spike train cross-correlation analysis. Example raw data show wide-band CA1 (upper trace, green; sharpwave-ripples marked by arrowheads) and PrL (lower trace, black) LFP during NREM sleep. Rasters show examples of single CA1 and PrL units, exemplifying firing patterns during ripples and spindles (**C1**) Histogram of spike widths (peak to trough) of all recorded PrL units. A bimodal Gaussian probability density function (PDF, grey line) was fitted to the histogram, with $\mu1 = 177.05$ μs and $\sigma1 = 16.64$ μs for the narrow spike width population, and $\mu2 = 301.48$ μs and $\sigma2 = 33.95$ μs for the wide spike width population (putative pyramidal cells). The cut-off for putative pyramidal cells was set to $(\mu2 - 2\sigma2) = 234$ μs, as indicated by the red line. (**C2**) Scatter plot of spike width against NREM firing rate, showing the 1Hz maximum firing threshold for putative pyramidal cells (dotted red line; Wierzynski et al. 2009). (**D1**) Cross-correlogram for the example units shown in **B** (bin size 10ms), using all spikes during a single recording session in the sleep box (red lines mark mean firing rate and 95% confidence estimates; black lines 100ms). (**D2)** Cross-correlogram for same cell pair using only spikes fired within 250ms of ripple peaks during NREM sleep.

Supplemental Table S1

Variable	CT	Lights	Control	n	MAM	n	change	t test t	t test p palue
Non-REM (% per hour)	0 - 5	ON	53.9 ± 1.5	14	44.0 ± 2.1	13	-9.9	4.25	**P<0.001**
	6 - 11	ON	47.8 ± 1.9	14	39.5 ± 2.0	13	-8.3	3.56	**P<0.01**
	12 - 17	OFF	22.8 ± 1.2	14	19.2 ± 1.4	13	-3.6	1.56	P > 0.05
	18 - 23	OFF	25.0 ± 1.5	14	18.7 ± 1.3	13	-6.2	2.68	**P < 0.05**
REM (% per hour)	0 - 5	ON	8.8 ± 0.5	14	6.9 ± 0.9	13	-1.9	2.08	P > 0.05
	6 - 11	ON	9.5 ± 0.7	14	8.2 ± 0.9	13	-1.4	1.47	P > 0.05
	12 - 17	OFF	4.9 ± 0.7	14	4.5 ± 0.7	13	-0.4	0.43	P > 0.05
	18 - 23	OFF	3.5 ± 0.3	14	2.9 ± 0.4	13	-0.6	0.61	P > 0.05
Wake (% per hour)	0 - 5	ON	37.3 ± 1.6	14	49.1 ± 2.7	13	11.8	4.18	**P<0.001**
	6 - 11	ON	42.7 ± 2.3	14	52.3 ± 2.5	13	9.6	3.41	**P<0.01**
	12 - 17	OFF	72.2 ± 1.6	14	76.2 ± 1.8	13	4.0	1.42	P > 0.05
	18 - 23	OFF	71.5 ± 1.8	14	78.3 ± 1.6	13	6.8	2.40	P > 0.05
Average sleep bout length (min)	0 - 5	ON	7.4 ± 0.7	14	3.8 ± 0.4	13	-3.6	6.29	**P<0.001**
	6 - 11	ON	4.9 ± 0.5	14	2.9 ± 0.3	13	-2.0	3.50	**P<0.01**
	12 - 17	OFF	2.9 ± 0.2	14	1.9 ± 0.1	13	-1.0	1.77	P > 0.05
	18 - 23	OFF	3.1 ± 0.3	14	1.9 ± 0.1	13	-1.2	2.06	P > 0.05
Average REM sleep bout length (min)	0 - 5	ON	1.5 ± 0.1	14	1.4 ± 0.1	13	-0.1	0.85	P > 0.05
	6 - 11	ON	1.5 ± 0.1	14	1.4 ± 0.1	13	-0.1	0.33	P > 0.05
	12 - 17	OFF	0.8 ± 0.1	14	0.9 ± 0.1	13	0.2	1.03	P > 0.05
	18 - 23	OFF	0.8 ± 0.1	14	0.8 ± 0.1	13	0.0	0.05	P > 0.05
Delta power (μV^2)	0 - 5	ON	0.100 ± 0.007	14	0.073 ± 0.007	13	0.0	3.87	**P<0.001**
	6 - 11	ON	0.075 ± 0.004	14	0.057 ± 0.005	13	0.0	2.38	P > 0.05
	12 - 17	OFF	0.074 ± 0.004	14	0.059 ± 0.005	13	0.0	2.09	P > 0.05
	18 - 23	OFF	0.087 ± 0.004	14	0.069 ± 0.005	13	0.0	2.40	P > 0.05
Sleep onset latency (from lights on)	CT0	ON	21.9 ± 1.8	14	21.3 ± 3.5	13	-0.6	1.20	P > 0.05
Time to first REM bout (from lights on)	CT0	ON	75.4 ± 8.0	14	81.6 ± 15.2	13	6.2	2.40	P > 0.05

Supplemental Experimental Procedures

EEG recordings

70-80 day old rats were anesthetized with isoflurane and prepared with a cranial implant consisting of five stainless steel screws, two located over motor cortex at +3.9mm AP, ± 2.0mm ML from bregma, two visual cortex −6.4mm AP, ±5.5mm ML from bregma, and one ground screw over the cerebellum. EMG was monitored via two Teflon-coated stainless steel wires under the nuchal trapezoid muscles. Body temperature (Tb) and locomotor activity (LMA) were monitored via abdominal miniature transmitters (PDT4000 E-Mitter, Minimitter, Bend, OR). Rats were housed individually within microisolator cages equipped with a custom low-torque slip-ring commutator (Hypnion, Inc., Lexington, MA), and hardware that permitted *ad libitum* access to food and water. Each Cage was located within a separate compartment of an electromagnetically shielded, ventilated, 16-animal stainless steel sleep recording chamber. Rats' circadian rhythms were entrained to a 24-h (LD 12:12) light-dark cycle (30–35 lux inside the cage).

Bipolar differential EEG recordings were amplified 10,000 times, filtered (0.3–30Hz) and digitized at 100Hz. In parallel with the differential EEG recording used for the sleep scoring, EEG signals from the contralateral motor and visual cortices were recorded and digitized at 2kHz (CED1401, Cambridge Electronic Design, UK) for spectral analyses. These separate EEG recordings were referenced to a cerebellar ground screw. Drinking and feeding activity were detected via infrared beam-break. All variables were monitored continuously and simultaneously.

Sleep scoring

Arousal states were determined using the automated sleep scoring algorithm based on SCORE™ that uses a combination of the salient features of the electroencephalogram and muscle tone (see Van Gelder et al., 1991). The process of assigning a state of wakefulness, TDW, REM or NREM sleep consisted firstly of extracting four features from the 10 second EEG epoch; amplitude, zero-crossings (a measure of EEG frequency), harmonic amplitude and harmonic frequency, these were then represented as 12 bin histograms. A mean of the integrated EMG over the 10s epoch along with the Boolean data from the LMA, drinking and feeding activity were also used in arousal state assignment. Unique templates were then generated from these parameters through a user-dependent teaching process; all users were blinded to treatment. The state scoring procedure was then performed using a pattern matching algorithm (explained in detail in Van Gelder et al., 1991), after exclusion of particular states on the basis of nonEEG data. REM sleep and NREM sleep for example, were excluded from consideration if the animals had moved or been drinking from its bottle during the epoch.

For analysis purposes, Wake and TDW were combined (WAKE). Data quality was assured by frequent on-line inspection of the signals. Sleep-wake scoring was scrutinized carefully for artefact by off-line visual examination of raw EEG waveforms and the distribution of integrated EMG values. EEG containing artefact was not used in EEG frequency analyses. All off line sleep analysis was performed blind with respect to MAM/SHAM treatment.

Tetrode Recordings

Six SHAM animals and five MAM animals were implanted with arrays of adjustable tetrode recording electrodes targeted to the PrL (+3.2 mm, +0.6 mm from bregma) and ipsilateral dorsal CA1 (−3.6 mm, +2.2 mm). Continuous LFP (sampled at 1KHz per channel, bandpass filtered between 0.1 and 475 Hz) and single unit activity (sampled at 32.5 kHz, filtered at 600Hz-6kHz) were recorded using a Digital Lynx recording system (Neuralynx, Bozeman, MT). Local reference electrodes were placed in a proximal cortical region without spiking activity (for PrL recordings) or in overlying white matter (CA1). A ground screw was also placed over the cerebellum. Electrolytic lesions established tetrode positions at the end of each experiment (Figure S4).

Sleep recordings were made immediately following exploration of a linear maze in order to ensure slow-wave- and spindle-rich sleep. Spindle rich NREM periods were easily identifiable by clear changes in the LFP to strong power in the spindle (10-15Hz) and delta (0.3-3Hz) frequencies. A spindle detection algorithm (see below) in combination with automated scoring of immobility from video allowed sleep periods to be identified and concatenated together. Only the first 10min of spindle rich sleep was used for the analysis to limit the sleep period to within the first hour after exploration of the maze.

Unit clustering and putative pyramidal cell identification

Raw threshold detected spikes were clustered using a combination of KlustaKwik (K.D. Harris, http://klustakwik.sourceforge.net/) and manual clustering (MClust; A.D. Redish, http://redishlab.neuroscience.umn.edu) based on spike energy, peak position and the first principal component of the waveform. Noise spikes were removed based on waveform features (waveform cutter). This yielded 100 units in PrL and 54 units in CA1 from 4 SHAM animals; and 70 units PrL and 44 units in CA1 from 4 MAM animals. Putative pyramidal cells were well-separated units with a mean NREM firing rate of 0.05-1 Hz and with a spike width larger than 234µs (FigureS4).

For spindle phase-locking analyses, a continuous phase was calculated from the Hilbert transform of one PrL LFP trace (band passed at 8-18Hz, least squares filter, EEGlab toolbox) and individual spike phase values were calculated by local linear interpolation of spindle phase. Only spikes within ±5s of spindle peaks and troughs larger than ±3.5 times the standard deviation of the entire signal were included. Rayleigh's test was used to assess a significant deviation from a uniform circular distribution of spike timing and the circular concentration coefficient (kappa) to quantify the strength of unit phase locking.

NREM cross-correlation analyses were adapted from Wierzynski et al. (2009) and are summarized in FigureS4. Putative pyramidal cell spike times from CA1 and PrL were filtered to select spikes that occurred within ±250ms of CA1 ripples; only units that fired at least 100 spikes during ripples were included. Cross-correlations were then calculated with 10ms bin size, and normalized by conversion to z-scores before peak cross-correlation values and time lags were detected.

Detection algorithms and spectral analysis

Individual EEG or LFP oscillatory events were detected using custom MATLAB (The MathWorks, Inc., Natick, MA) routines. All EEG or LFP traces were first band pass filtered (slow-waves: 0.3-3 Hz, spindles: 8-18 Hz, ripples: 120-250 Hz; least squares filtering, EEGlab toolbox, Delorme and Makeig, 2004) and then transformed to z-scores.

Delta waves were detected directly from the filtered, z-score signal with a threshold of 3.5 times the SD of the whole signal in both negative and positive direction. Only waves that contained a negativity larger than 80µV were included. The criteria for delta wave detection were applied independently to both motor and visual cortical electrodes, used for all animals and resulted in a reproducible and specific detection of high-amplitude slow-waves that occurred during late sleep in each bout.

For detection of cortical spindles and hippocampal ripples the z-score of the respective filtered signal was rectified and the envelope was determined using a cubic spline interpolation between the maxima of the rectified signal. This envelope was then used for detection of threshold crossings. Spindles were detected using a threshold of 3.5 SD of the signal. Start and finish times of the spindle were also calculated (2 SD of the signal). Spindles were rejected if they were shorter than 500ms or longer than 2s; spindles occurred within 100ms of one another were treated as one event. Ripples were detected using a threshold of 3.5 SD (2 SD for start and end times) and rejected if they were shorter than 50ms or longer than 500ms. If gaps of less than 50ms occurred between ripples, they were treated as one event. All automatic detection procedures were validated and parameters were tuned through independent visual scoring of the EEG/LFP.

Multi-taper spectral analysis was used to calculate power spectra and coherence for LFP data between the two cortical recording locations (Chronux toolbox, www.chronux.org). To investigate the temporal relationship between hippocampal ripples and spindle events on a slow time scale, cross-correlation between the respective powers was computed. Spectral power in these frequency bands was calculated in overlapping 1-s windows, providing a time series, which were log-transformed and cross-correlated.
